# Chemistry of Secondary Polyphenols Produced during Processing of Tea and Selected Foods

**DOI:** 10.3390/ijms11010014

**Published:** 2009-12-28

**Authors:** Takashi Tanaka, Yosuke Matsuo, Isao Kouno

**Affiliations:** Laboratory of Natural Product Chemistry, Graduate School of Biomedical Sciences, Nagasaki University, 1-14 Bunkyo-machi, Nagasaki 852-8521, Japan; E-Mails: y-matsuo@nagasaki-u.ac.jp (Y.M.); ikouno@nagasaki-u.ac.jp (I.K.)

**Keywords:** polyphenol, oxidation, black tea, catechin, proanthocyanidin, ellagitannin

## Abstract

This review will discuss recent progress in the chemistry of secondary polyphenols produced during food processing. The production mechanism of the secondary polyphenols in black tea, whisky, cinnamon, and persimmon fruits will be introduced. In the process of black tea production, tea leaf catechins are enzymatically oxidized to yield a complex mixture of oxidation products, including theaflavins and thearubigins. Despite the importance of the beverage, most of the chemical constituents have not yet been confirmed due to the complexity of the mixture. However, the reaction mechanisms at the initial stages of catechin oxidation are explained by simple quinone–phenol coupling reactions. *In vitro* model experiments indicated the presence of interesting regio- and stereoselective reactions. Recent results on the reaction mechanisms will be introduced. During the aging of whisky in oak wood barrels, ellagitannins originating from oak wood are oxidized and react with ethanol to give characteristic secondary ellagitannins. The major part of the cinnamon procyanidins is polymerized by copolymerization with cinnamaldehyde. In addition, anthocyanidin structural units are generated in the polymer molecules by oxidation which accounts for the reddish coloration of the cinnamon extract. This reaction is related to the insolubilization of proanthocyanidins in persimmon fruits by condensation with acetaldehyde. In addition to oxidation, the reaction of polyphenols with aldehydes may be important in food processing.

## 1. Introduction

Recent studies have revealed various health benefits of plant polyphenols, and their importance in foods, beverages and natural medicine [1–7]. The polyphenols include different types of chemical compounds including catechins, proanthocyanidins, anthocyanins, gallotannins, ellagitannins, flavonol glycosides, hydroxycinnamoyl esters, lignoids, and stilbenoids. These subclasses of polyphenols show different chemical reactivities. The polyphenols are stable as long as they are accumulated in living plant cells. However, when the tissues undergo physiological changes, such as fruit ripening and wounding of the tissue by herbivores, some of the polyphenols are chemically converted to secondary polyphenols by enzymatic and non-enzymatic reactions. A typical and economically important example of secondary polyphenols is black tea polyphenols. During black tea production, fresh tea leaves are cut and kneaded. At this stage catechins and oxidation enzymes, which are stored in different tissues in the leaf, are mixed together and the catechins are converted into a complex mixture of oxidation products. Among plant secondary metabolites, polyphenols are most susceptible to oxidation and their reactivity is closely related to plant defense systems against oxidative stress. Therefore, post harvest chemical change of polyphenols occurs ubiquitously in vegetables and fruits to a greater or lesser extent. In most cases, the reactions involving the production of secondary polyphenols are complex, and many of the reaction products still remain to be chemically identified. However, the reaction mechanisms are interesting and attractive from the viewpoint of natural product chemists. This review describes the production mechanisms of black tea polyphenols from simple tea catechins. In addition, some reactions of polyphenols with coexisting compounds such as various aldehydes are also introduced.

## 2. Secondary Polyphenols Produced by Oxidation

### 2.1. Oxidation of (+)-catechin and (−)-epicatechin

**Figure 1.**
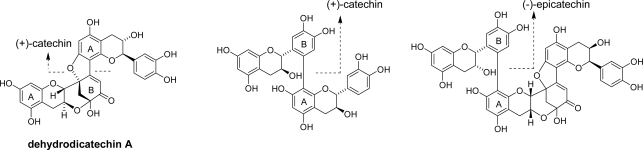
Structures of dehydrodicatechin A and related compounds [8–10].

**Scheme 1.**
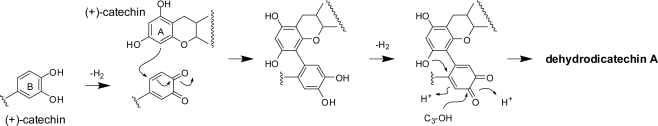
Production of dehydrotheasinensin A from (+)-catechin.

Enzymatic or non-enzymatic oxidation is the most common reaction involved in the production of secondary polyphenols. Polyphenols having *ortho*-diphenol (catechol) aromatic rings are widely distributed in nature and susceptible to oxidation. The structure of dehydrodicatechin A (Figure 1) was first reported in 1969 [8], and generation of related catechin dimers and trimers by *in vitro* experiments followed [9,10]. These compounds are produced by nucleophilic addition of electron-rich phloroglucinol A-rings to the electron-deficient *ortho*-quinone of B-rings. In the case of dehydrodicatechin A, further oxidation of the catechol ring and addition of hydroxyl groups to a double bond and a ketone occurs (Scheme 1). These types of oxidation products are partly responsible for the browning of fruits and beer [11]. The *in vitro* oxidation of (+)-catechin *via* an oxidation enzyme usually yields a complex mixture, including oligomers, because each catechin molecule has three reaction sites: the C-6 and C-8 of the A-ring and the C-6 of the B-ring [12]. Catechin oxidation products related to these compounds have also been isolated from *Quercus ilex* [13] and some crude drugs [14,15].

### 2.2. Black Tea Polyphenols

The polyphenols from fresh tea leaf are quite unique. Their concentrations in the leaf are very high (13–25% of dry weight [16]) and most of the polyphenols are composed of only four monomeric catechins, that is, (−)-epicatechin (EC), (−)-epicatechin-3-*O*-gallate (ECg), (−)-epigallocatechin (EGC), and (−)-epigallocatechin-3-*O*-gallate (EGCg). Commonly, monomeric catechins coexist with proanthocyanidins in many other plants; however, the concentration of proanthocyanidins in the tea leaf is low. Coexistence of pyrogallol (3,4,5-tryhydroxyphenyl)- and catechol (3,4-dihydroxyphenyl)-type catechins is also characteristic of tea leaf polyphenols. In addition, over 50% of the catechins are esterified with gallic acid and EGC and EGCg together account for over 70% of the total tea catechins [16]. The polyphenol composition of commercial green tea consumed mainly in East Asia is similar to that of fresh tea leaves because steaming or roasting at the initial stage of green tea production inactivates enzymes involved in the oxidation and hydrolysis of the chemical constituents of the leaves. In contrast, the enzymes play important roles in black tea manufacturing. The complex enzymatic reactions produces the color and flavor characteristic of each black tea brand. As for polyphenols, when fresh green tea leaves are crushed at the initial stage, the four major catechins are enzymatically oxidized.

**Scheme 2.**
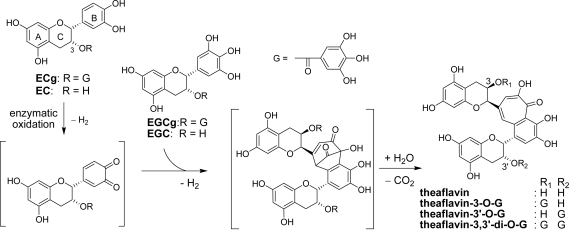
Production of theaflavins from epicatechin and epigallocatechin [17,20].

Oxidation of a mixture of these four catechins in the tea leaf proceeds in different ways from that observed for (+)-catechin alone. Coupling products between A- and B-rings as observed in the oxidation of (+)-catechin have not been found so far in black tea polyphenols. The most important catechin oxidation products in black tea are theaflavin and its mono and digallates [17–19]. Theaflavin possesses a characteristic benzotropolone moiety, which is produced by condensation between a catechol-type B-ring of EC and a pyrogallol-type B-ring of EGC. The reaction mechanism was presumed to be as shown in Scheme 2 [20]. *In vitro* oxidation of a mixture of EC and EGC with polyphenol oxidase suggested that the enzymes preferentially oxidize EC to EC-quinone, and the electron deficient EC-quinone reacted with the electron-rich EGC B-ring. Subsequent oxidation and decarboxylation afforded theaflavin. During the reaction, generation of a bicyclo[3.2.1]octane-type intermediate as shown in Scheme 2 was presumed [17,20].

**Figure 2.**
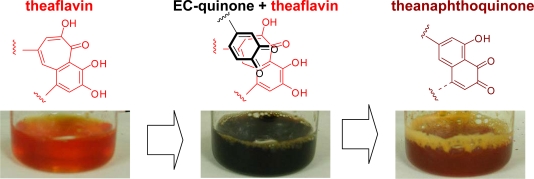
Color changes during enzymatic oxidation of EC and EGC [10].

**Scheme 3.**
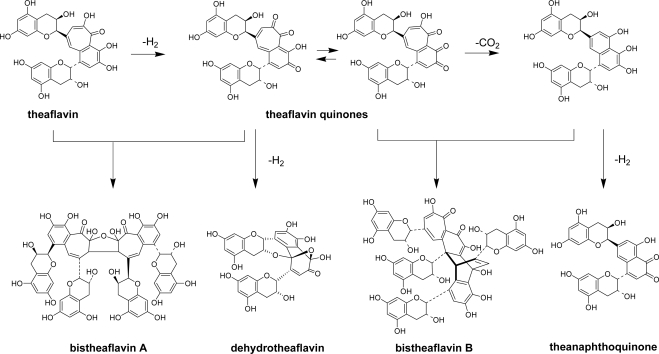
Oxidation of theaflavins [21–24].

Theaflavins are not final products and thus, are further oxidized (Scheme 3). EGC was consumed faster than EC, because, as described below, oxidative coupling between two molecules of EGC occurs. When EGC is exhausted in the reaction mixture, the EC-quinone begins to oxidize theaflavin. At that time, the brilliant reddish orange color of the reaction mixture dramatically changes to dark green [10] (Figure 2). This is probably caused by stacking of the EC-quinone on the benzotropolone ring of the theaflavins. The oxidation of theaflavins proceeds *via* the electron withdrawing action of the EC-quinone from the benzotropolone ring. Several oxidation products of the theaflavins are known. The major product is theanaphthoquinone [21,22]. However, this product has not been isolated from commercial black tea to date. In the actual black tea fermentation, coexisting substances may react with theaflavin quinones or theanaphthoquinone. Dehydrotheaflavin, bistheaflavins A and B were also only isolated as *in vitro* oxidation products of EC and EGC [23,24].

**Figure 3.**
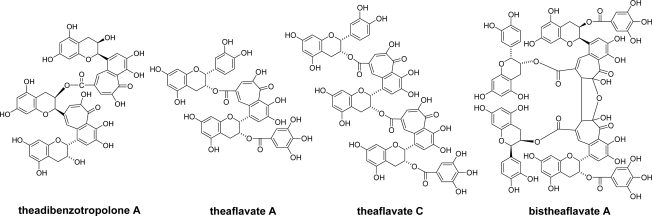
Structures of oxidation products produced by condensation between catechol-type B-rings and galloyl groups [25–30].

Compared with B-rings, the reactivity of the galloyl esters is low. However, oxidative coupling between EC-quinone and the galloyl groups of the theaflavin gallates has been observed (Figure 3) [25–27]. The reaction elongates the molecules of oxidation products and may be related to the formation of polymeric oxidation products. In tea fermentation, ECg is less reactive compared with other catechins and its concentration decreases slowly. However, enzymatic oxidation of ECg alone yielded condensation products with a benzotropolone ring produced by coupling between galloyl groups and the catechol B-rings [28–30]. The coupling products between the A- and B-rings as observed in the oxidation of (+)-catechin or EC (Figure 1) were not isolated.

Pyrogallol-type B-rings of EGC and EGCg have the lowest redox potential among the aromatic rings of tea catechins [31] and are susceptible to oxidation. In addition to direct oxidation with enzymes, the pyrogallol rings are oxidized by EC-quinone. Importance of the EC-quinone and related catechol-quinones, such as chlorogenic acid quinone, as an oxidizing agent in pyrogallol oxidation is supported by the oxidation of myricitrin [32]. Enzymatic oxidation of myricitrin alone is very slow. However, myricitrin was oxidized rapidly in the presence of (+)-catechin or chlorogenic acid.

**Figure 4.**
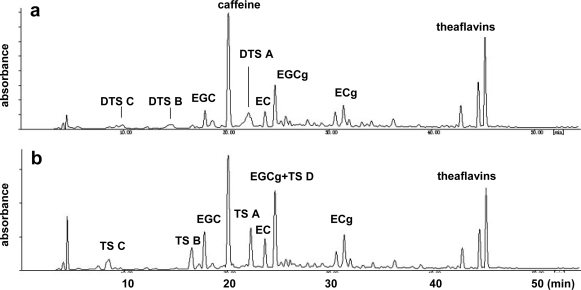
HPLC of crushed tea leaf (a) before heating (b) after heating (80 °C). DTS: dehydrotheasinensins, TS: theasinensins [34].

The oxidations of EGC and EGCg are important because these two catechins account for over 70% of total tea catechins in tea leaves. The following *in vitro* experiments demonstrate that the production of unstable quinone dimers named dehydrotheasinensins is the most important reaction in the oxidation of these catechins [33,34]. After the fresh tea leaves were crushed and kneaded, theaflavins were produced in the leaves; however, theasinensins, which are also major black tea polyphenols, were not detected [35,36]. Production of theasinensins was only observed after the leaves were heated at 80 °C (Figure 4).

**Scheme 4.**
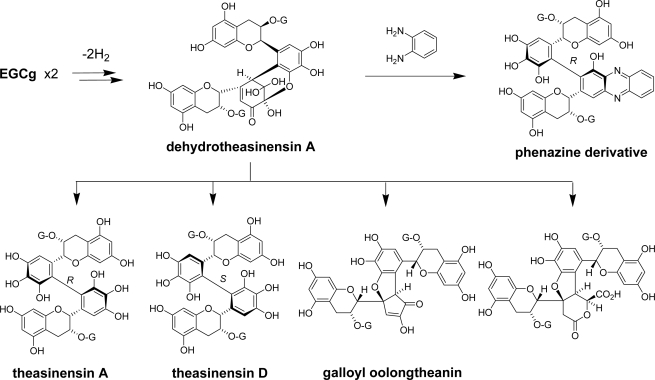
Production and decomposition of dehydrotheasinensin A [33].

The results indicated that the theasinensins are produced by degradation of heat-susceptible intermediates. The presence of the intermediates was first confirmed by trapping them as phenazine derivatives by condensation with *o*-phenylenediamine. The structure of the derivatives indicated that the unstable intermediates are quinone dimers of EGC and EGCg. The total concentration of the intermediates in the leaves was estimated to be comparable to that of theaflavins. One of the intermediates was synthesized and purified by *in vitro* enzymatic oxidation of EGCg and named dehydrotheasinensin A [33]. This compound has a hydrated form of a triketone structure. Although there are two isomers of an EGCg dimer: one is theasinensin A with a *R*-biphenyl bond and the other is theasinensin D with a *S*-biphenyl bond [36], reduction of dehydrotheasinensin A with thiol compounds, such as mercaptoethanol, gave only theasinensin A. The results indicated that oxidative coupling between two EGCg molecules proceeds stereoselectively. In contrast, dehydrotheasinensin A slowly decomposed in a neutral aqueous solution to give a mixture of theasinensins A and D, galloyl oolongtheanin [36], and another oxidation product having a carboxylic acid. Theasinensins are reduction products and other two are oxidation products; therefore, the production of theasinensins from dehydrotheasinensin A proceeds vis a redox dismutation (Scheme 4). However, concentration of theasinensin A in black tea is higher than that of theasinensin D and galloyl oolongtheanin, suggesting that dehydrotheasinensins are reduced by some reducing substances, such as ascorbic acid, present in the tea leaf. It should be noted that dehydrotheasinensin A is not produced by enzymatic oxidation of theasinensin A [37].

**Scheme 5.**
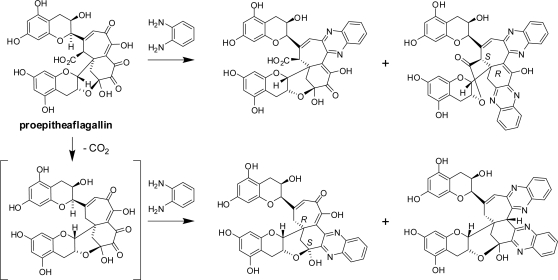
Quinoxaline derivatives of proepitheaflagallin [38].

**Scheme 6.**
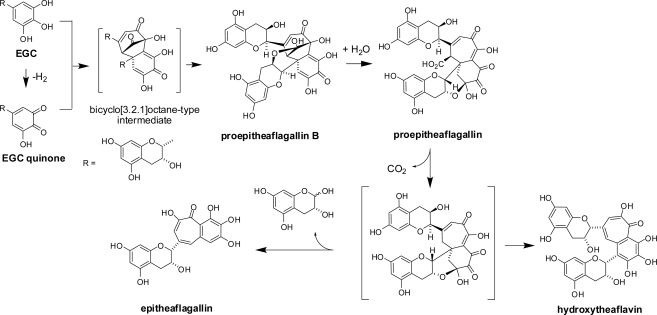
Production and decomposition of proepitheaflagallin [38,40].

The major oxidation product of EGC is dehydrotheasinensin C, a desgalloyl form of dehydrotheasinensin A, which decomposes to give theasinensins C and E and desgalloyl oolongtheanin, which are desgalloyl analogs of the products generated from dehydrotheasinensin A. Oxidation of EGC is partly different from that of EGCg, and a characteristic oxidation product named proepitheaflagallin was isolated (Scheme 5) [38]. In addition to the usual 2D NMR spectroscopic analysis, the structure of proepitheaflagallin was determined by analysis of the condensation products with *o*-phenylenediamine. The structures of the quinoxaline derivatives indicated that proepitheaflagallin exists as a mixture of several tautomers. Occurrence of decarboxylation was also noted. In production of proepitheaflagallin, the free hydroxyl group at C-3 of the flavan-3-ol skeleton plays an important role by forming an acetal ring with the B-ring carbonyl carbons. This product is unstable and degraded to give the known black tea pigment epitheaflagallin (Scheme 6) [39].

In addition to epitheaflagallin, hydroxytheaflavin was also produced by the degradation. This product has not been identified in commercial black tea. Recently, production of proepitheaflagallin B having a bicyclo[3.2.1]octane-type structure was demonstrated by *in vitro* oxidation of EGC [40]. This unstable compound decomposed to afford proepitheaflagallin. Interestingly, structure of the intermediate was related to that presumed in theaflavin synthesis (Scheme 2). Formation of a hemiacetal ring between the C-3 hydroxyl and carbonyl groups in proepitheaflagallin B stabilizes the molecule under conventional chromatographic conditions. Isolation of this product provides the first substantial evidence that a bicyclo[3.2.1]octane-type intermediate is produced during catechin oxidation.

**Scheme 7.**
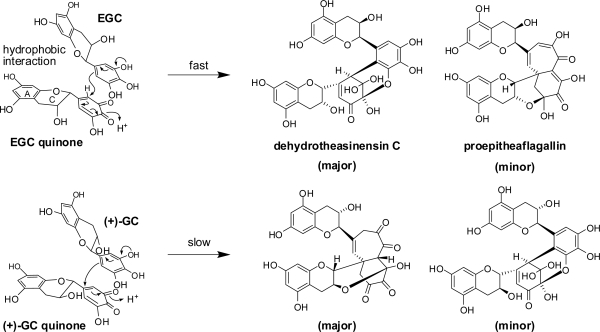
Difference of oxidative coupling of EGC and GC [41].

Interestingly, oxidation of EGC with a 2(*R*),3(*R*)-*cis* configuration and (+)-gallocatechin (GC) with a 2(*R*),3(*S*)-*trans* configuration proceeds differently [41], and enzymes preferentially oxidize EGC rather than GC. Oxidation of EGC proceeds rapidly and, as mentioned above, the major oxidation product is dehydrotheasinensin C while proepitheaflagallin is the minor product. In contrast, oxidation of (+)-GC is slow and the major product is a 2,3-*trans* analog of proepitheaflagallin (Scheme 7). Only a small amount of the dehydrotheasinensin-type product was detected, even though a substantial amount of GC quinone was present in the reaction mixture. This was probably because the C-3 hydroxyl group hinders the intermolecular interaction between GC and GC quinone. The stereochemistry of the C-ring also affected the regioselectivity of the coupling. In the oxidation of EGC, the hydrophobic interaction between two sets of the A- and C-rings may accelerate the coupling reaction. Oxidation of (−)-GC was similar to that of (+)-GC, suggesting that the enzyme only oxidized the B-ring to *o*-quinone, and the subsequent dimerization reaction was non-enzymatic.

**Scheme 8.**
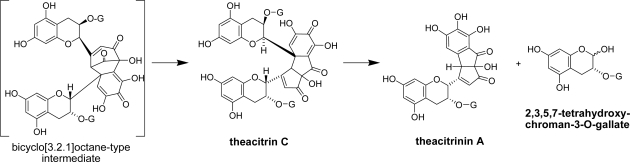
Production and decomposition of theacitrin C [42,43].

Davis *et al*. demonstrated the presence of the yellow pigments theacitrins A, B and C in commercial black tea. These pigments are also produced *via* the bicyclo[3.2.1]octane intermediates [42]. Recently we found that theacitrin C decomposes to give a monomeric pigment named theacitrinin A together with 2,3,5,7-tetrahydroxychroman-3-*O*-gallate [33,43]. This reaction mechanism is related to that of the production of epitheaflagallin from proepitheaflagallin (Scheme 8) and the presence of the tetrahydroxychroman gallate in commercial black tea was confirmed [33].

**Figure 5.**
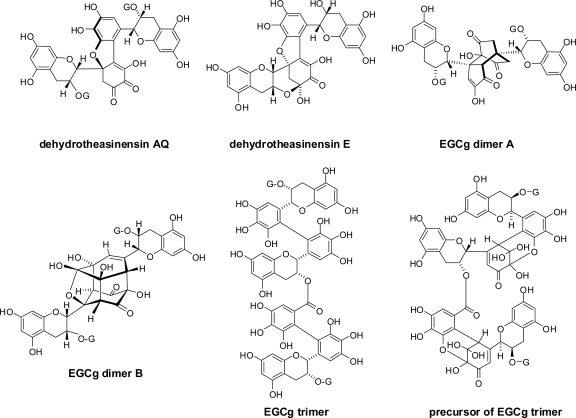
Structures of oxidation products produced from EGCg [23,37,44].

Some oxidation products related to theasinensins were produced by *in vitro* enzymatic oxidation of EGC and EGCg (Figure 5). Dehydrotheasinensin AQ [44] and dehydrotheasinensin E [10] are yellow pigments and deduced to be produced by isomerization of dehydrotheasinensins A and C, respectively. Dehydrotheasinensin AQ was detected in commercial black tea.

EGCg dimer A was first isolated as an *in vitro* oxidation product of EGCg and later isolated from commercial black tea [23]. EGCg dimer B was isolated from a mixture after treatment of EGCg oxidation products with mercaptoethanol as a reducing agent of quinones [44]. A trimer of EGCg was also produced by oxidative coupling between the EGCg B-ring and the galloyl group of theasinensin A [44]. The coupling proceeded *via* a dehydrotheasinensin-type intermediate; however, the coupling is not stereoselective [37]. So far, EGCg trimers have not been found in black tea. *In vitro* experiments indicated that the reactivity of galloyl groups is much lower than that of pyrogallol and catechol B-rings.

When thinking about the health benefits of black tea polyphenols as antioxidant [45], anticancer [46,47] and anti-inflammatory [48,49] agents, it should be considered that the direct absorption of the polyphenols by the digestive tract is expected to be lower than for tea catechins [50] because the black tea polyphenols, represented by the theaflavins and theasinensins, have larger molecular weights than those of monomeric tea catechins. In contrast, the inhibition of digestive enzymes may have considerable importance, since polyphenols with high molecular weights have an inherent ability to interact with proteins by forming hydrophobic and hydrogen bonds [51,52] which usually results in enzyme inhibition [53]. Amylase and lipase are digestive enzymes that hydrolyze starch and triglyceride, respectively, and inhibition of these enzymes has been linked to the decreased incidence of common diseases caused by diets rich in carbohydrates and fat. The inhibitory activities of theaflavins against these enzymes have been reported [54–56]; however, the concentrations of theaflavins in black tea infusions were much lower than other water-soluble polyphenols [20,57].

**Figure 6.**
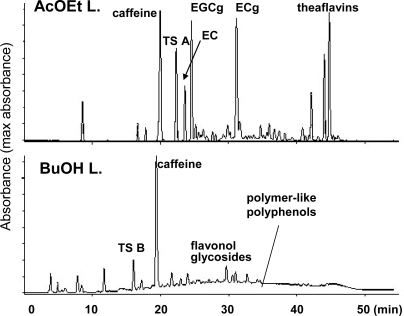
HPLC of AcOEt and n-BuOH layers of black tea extract [58].

Activity guided separation of black tea extract indicated that polymer-like polyphenols, in addition to theaflavins, have strong inhibitory activities towards lipase [58]. The uncharacterized polyphenols are detected as a broad hump on the HPLC baseline and are probably identical to the thearubigins (Figure 6). Thearubigins are major components of the color of black tea infusions [59,60], which are known to be heterogeneous mixtures of catechin oxidation products and have not yet been chemically characterized [57]. The ^13^C-NMR spectrum of the polymeric substance showed signals attributable to flavan-3-ol A- and C-rings and galloyl groups. Absence of the B-ring carbon signals suggested that the polymerization occurred at the B-rings. Recently, conjugation of catechin quinones with proteins was suggested [61]. The elemental analysis indicated that the nitrogen content of the polymeric substance is much lower (less than 0.3%) than that expected for protein conjugation. The mechanism of polymerization is still ambiguous.

Many plants have the ability to oxidize catechins even though the plants do not contain catechins [10]. When pure tea catechins are mixed with Japanese pear or loquat fruits homogenates, theaflavins, theasinensins and thearubigins are produced [10,33,37,44]. Oxidation of polyphenols is accompanied by reduction of an oxygen molecule to generate the superoxide anion and hydrogen peroxide, which are known to show antimicrobial activity [62–65]. In plant defense systems, generation of these reactive oxygen species plays important roles including the precipitation of proteins and enzyme inhibition in the damaged tissue *via* the resulting oxidation products of polyphenols. Production of hydrogen peroxide in tea leaf during enzymatic oxidation may also play an important role in the antimicrobial activity [66,67]. From the autoxidation of EGCg, production of dehydrotheasinensin A was also observed [33].

During black tea production, enzymatic oxidative dimerization of pyrogallol-type catechins is important because of their high susceptibility to oxidation and abundance in the tea leaf. Production of unstable intermediates, such as dehydrotheasinensins and proepitheaflagallin, was demonstrated. However, degradation of the intermediates remained to be clarified as the major degradation products, such as the theasinensins, only account for about half of the total degradation products. The unknown degradation products may hold the key to the solution of thearubigin formation in black tea chemistry.

### 2.3. Whisky

**Scheme 9.**
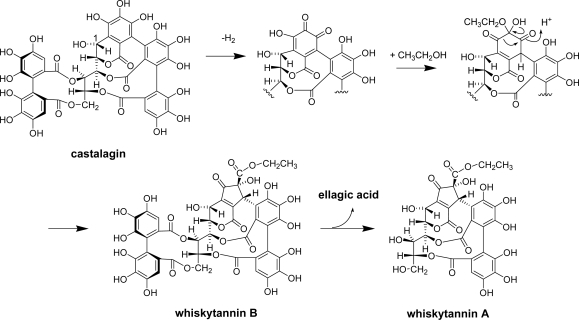
Production of whisky tannins A and B from castalagin [70].

In whisky production, distilled spirits are aged for several years in oak barrels, and the constituents of the wood dissolve into the spirit to determine its color, flavor and taste. The wood of oak species, such as *Quercus robur*, *Q. petraea*, and *Q. alba*, contains significant amounts of ellagitannins [68]. During barrel production, the oak wood passes through several processing stages, including seasoning and toasting processes, and the ellagitannin composition of the wood undergoes various chemical changes [69]. Therefore, the solute of the whisky is different from the original oak wood polyphenols. The major ellagitannins are C-glycosidic ellagitannins, castalagin and vescalagin and their dimers and oligomers [68]. These tannins decompose during the toasting or charring process. In addition, during the aging process, oxygen molecules penetrate into the spirits through the barrel wood and oxidize the solutes. Therefore, the polyphenols in whisky are a mixture of products generated through a complex chemical process. Recently, oxidation products of castalagin named whiskytannins A and B were isolated from commercially bottled Japanese whisky along with carboxyl ellagic acid, gallic acid, ellagic acid, brevifolin carboxylic acid, 6-*O*-and 2,3-di-*O*-galloyl glucoses, 2,3-(*S*)-hexahydroxy-diphenoylglucose, and castacrenin B [70]. In this experiment, castalagin and vescalagin were not detected. The structure of whisky tannins suggested that they are generated by regioselective oxidation of the pyrogallol ring attached to the glucose C-1 of castalagin and subsequent addition of ethanol and benzylic acid-type rearrangement (Scheme 9). It was reported that in the ethanol solution of vescalagin, a C-1 epimer of castalagin was converted to β-1-*O-*ethylvescalagin [71]; however, this ethanol adduct was not isolated from Japanese whisky.

**Figure 7.**
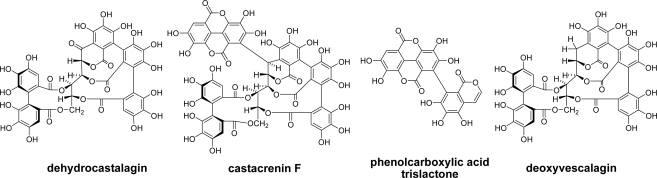
Pyrolysis products of castalagin and vescalagin [70,72].

To mimic the oxidation occurring during the charring process of barrel making, pyrolysis of ellagitannins was examined. Pyrolysis of castalagin, as a mimic of decomposition during the charring process in barrel production, yielded ellagic acid, dehydrocastalagin, castacrenin F, and phenolcarboxylic acid trislactone having an isocoumarin structure (Figure 7) [70,72]. Interestingly, pyrolysis of vescalagin afforded the deoxy product instead of the oxidation product [72].

## 3. Secondary Polyphenols Produced by Reaction with Coexisting Compounds

### 3.1. Oolong Tea and Black Tea

**Scheme 10.**
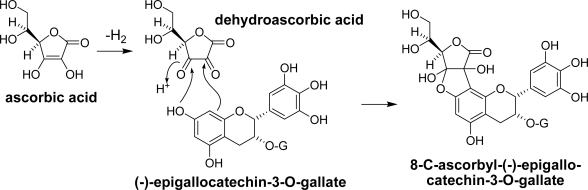
Formation of 8-C-ascorbyl-(−)-epigallocatechin-3-*O*-gallate [73].

During the tea fermentation process, coupling of the catechin A-ring with coexisting carbonyl compounds occurs. 8-C-Ascorbyl-(−)-epigallocatechin-3-*O*-gallate (Scheme 10) was first isolated from oolong tea, which is a kind of semi-fermented tea [73]. This compound is a coupling product between EGCg and dehydroascorbic acid.

**Figure 8.**
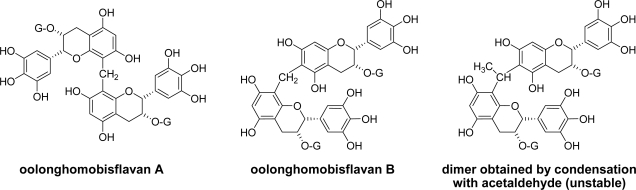
Structures of oolonghomobisflavans and an unstable dimer of EGCg produced by reaction with acetaldehyde [73,74].

EGCg dimers produced by reaction with formaldehyde were also isolated from the oolong tea, and named oolonghomobisflavans [73]. Usually, the C-C bond between the aldehyde and A-rings are unstable because nucleophilic substitution at the methine or methylene carbon between two phenyl groups easily occurs. Oolonghomobisflavans (Figure 8) are relatively stable compared with the analogous EGCg-acetaldehyde coupling products [74,75].

**Scheme 11.**
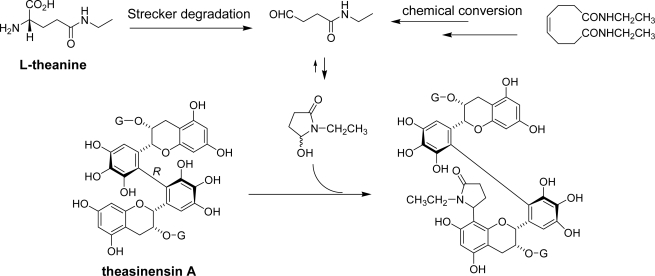
Reaction of theasinensin A with theanine Strecker aldehyde [80].

Aldehydes are also produced from amino acids by Strecker degradation in the presence of carbonyl compounds. Tea contains a characteristic amino acid named l-theanine (5-*N*-ethylglutamine) accounting for over 50% of the total amino acids of the tea leaves. The concentration of theanine is known to decrease during tea fermentation [76,77]. The catechin quinones possibly react with amino acids and generate Strecker aldehydes [78,79]. Evidence of the production of theanine Strecker aldehyde during tea fermentation was obtained from commercial black tea as a coupling product with theasinensin A [80]. The structure was confirmed by semisynthetic of the coupling product by preparation of 1-ethyl-5-hydroxy-2-pyrrolidinone, a cyclic form of the Strecker aldehyde, and subsequent coupling with theasinensin A (Scheme 11). 1-Ethyl-5-hydroxy-2-pyrrolidinone and related adducts of monomeric catechins were not detected in the black tea. Actually, attempts to produce a similar adduct with EGCg under the same reaction conditions failed. In the case of theasinensin A, the C-C bond between pyrrolidinone and the phloroglucinol A-ring was probably stabilized by the presence of another EGCg unit.

### 3.2. Insolubilization of Proanthocyanidins in Persimmon Fruits

**Scheme 12.**
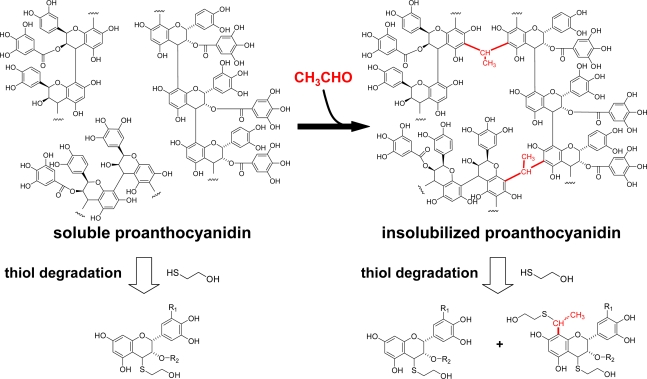
Insolubilization of proanthocyanidins in persimmon fruits [74].

Some plants use animals to disperse their seeds in exchange for delicious and nutritious fruit flesh. In the case of persimmon fruits, until the seeds acquire germinating ability, the fruits are protected by the bitter and astringent taste of proanthocyanidins. After the seeds acquire the germinating ability, the astringency decreases and the color of the fruits change to reddish orange. At this stage, acetaldehyde is secreted from the seeds and penetrates into the tannin cell [81]. The acetaldehyde concentration also artificially increases by treatment of the astringent persimmon fruit with ethanol or carbon dioxide under anaerobic conditions [75,82]. Acetaldehyde reacts with C-8 or C-6 of the proanthocyanidin A-rings and connects two proanthocyanidin molecules resulting in their insolubilization and a decrease in astringency (Scheme 12). The covalent bonding of acetaldehyde in insolubilized proanthocyanidins was supported by thiol degradation experiments, which degrade proanthocyanidins into their component flavan-3-ol units. [74]. Thiol degradation of the extract of the astringent fruits with mercaptoethanol-HCl afforded the thioethers of flavan-3-ols. However, after the persimmon fruits were treated with ethanol under anaerobic conditions, the extract of the fruits did not yield the thio ethers. Direct treatment of the plant debris remaining on the filter paper with mercaptoethanol-HCl afforded the bisthioethers of the flavan-3-ol acetaldehyde adducts in addition to the usual thioethers.

### 3.3. Cinnamon Bark

**Figure 9.**
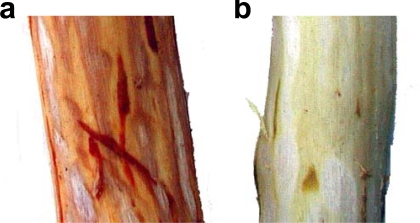
Surface of Japanese cinnamon wood after the bark was peeled off. (a) Fresh cinnamon (b) cinnamon heated with microwave oven for 30 seconds [83].

Reaction of proanthocyanidins with aldehydes has also been observed when plant tissues are wounded. When the fresh bark of Japanese cinnamon (*C. sieboldii*) was peeled from a branch, the color of the wood surface was immediately changed from white to reddish brown (Figure 9). Since this dynamic change in color was not observed when the branch was heated in advance, the reaction was apparently catalyzed by enzymes.

**Scheme 13.**
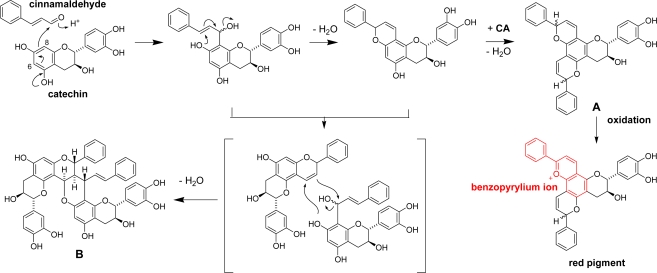
Reaction of (+)-catechin and cinnamaldehyde and generation of red pigment [83].

We found that a similar color change was also observed when a mixture of (+)-catechin and cinnamaldehyde, a dominant essential oil of the cinnamon bark, was heated at 100 °C [83]. Even at room temperature, the color changed to red very slowly to give a complex mixture of condensation, monomeric (A) and dimeric (B) products (Scheme 13). The monomeric product A is susceptible to autoxidation to give a red pigment containing benzopyrylium ion moieties which are related to anthocyanidins pigments. On the surface of cinnamon wood (Figure 9), the oxidation of the procyanidin-cinnamaldehyde conjugates is probably catalyzed by oxidation enzymes.

**Figure 10.**
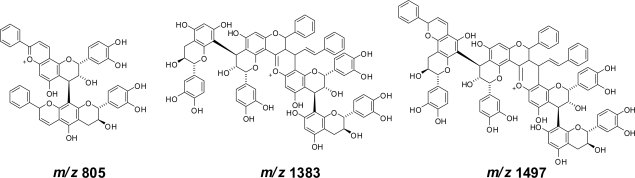
Possible structures of the fragment ion peaks observed in the MALDI-TOF MS of the reaction products of procyanidin B1 and cinnamaldehyde [83].

The production of dimeric product B indicated that dimerization and oligomerization of procyanidins occurs. This was supported by the MALDI-TOF MS of the reaction products of procyanidin B1 [(−)-epicatechin-(4β→8)-(+)-catechin] and cinnamaldehyde (Figure 10). Production of the benzopyrylium ion was suggested by the appearance of the ion peaks and color of the reaction mixture. The condensation of the proanthocyanidins with cinnamaldehyde in the cinnamon extract was confirmed by a ^13^C-NMR spectrum of polymeric proanthocyanidins obtained by size-exclusion chromatography [84], which showed signals arising from the phenyl groups of the cinnamaldehyde units.

### 3.4. Application of Catechin-aldehyde Conjugation

**Figure 11.**
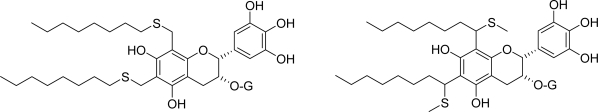
Structures of hydrophobic derivatives of EGCg [89,90].

It is well known that tea catechins show strong radical scavenging activities [85–88]. However, they are hydrophilic compounds and do not dissolve in a lipid layer. Some efforts have been made to synthesis lipid soluble derivatives of tea catechins by applying the reaction of EGCg with formaldehyde or alkylaldehyde and then subject the products to nucleophilic substitution with thiol compounds [89,90] (Figure 11).

**Figure 12.**
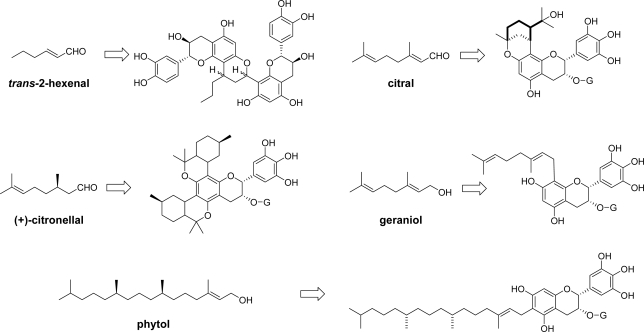
Structures of the conjugates of catechins with naturally occurring aldehydes [91].

Recently, the reaction of a conjugated aldehyde with a C-8(6) carbon and C-7(5) hydroxyl group of flavan-3-ols was applied to prepare lipid-soluble derivatives of catechins [91]. In addition to naturally occurring conjugated aldehydes (*trans*-2-hexenal and citral), a non-conjugated unsaturated aldehyde (citronellal) and allyl alcohols (geraniol and phytol) are also afforded products as shown in Figure 12. Although most of the reactions gave complex mixtures, the triglyceride soluble fractions of the reaction mixture of EGCg and citronellal, geraniol and phytol showed strong radical scavenging activities, ten times stronger than that of the triglyceride fraction of EGCg.

### 3.5. Reaction of Polyphenols with Aldehyde in Other Foods

**Figure 13.**
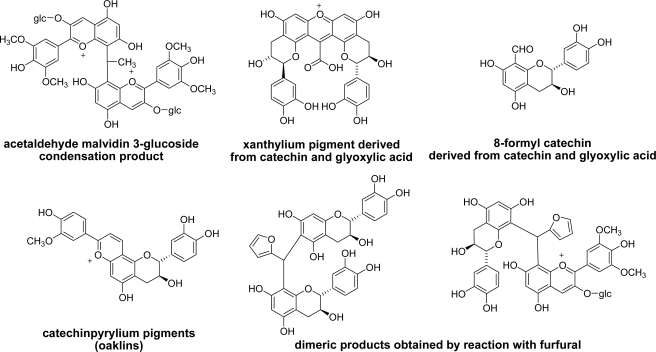
Structures of a condensation product of acetaldehyde and malvidin 3-glucoside, two glyoxylic acid-catechin condensation products, a catechinpyrylium product (oaklin) obtained by reaction with coniferyl aldehyde, and dimeric products produced by reaction with furfural in wine-like model solution.

Polyphenols in red wine undergo complex reactions with coexisting substances. Dimerization and polymerization of catechin, procyanidin, and anthocyanins in the presence of various aldehydes have been demonstrated in wine-like model solutions [92]. Condensation of malvidin 3-*O*-glucoside and acetaldehyde affords dimeric pigments [93] (Figure 13). Reaction of catechin with glyoxylic acid yields characteristic pigments with a xanthylium chromophore [94] and 8-formyl catechin [95]. In addition, several pigments named oaklins were generated by the reaction between catechin and coniferyl aldehyde or sinapyl aldehyde extracted from oak wood [96]. These aldehydes are produced by degradation of oak wood lignin and extracted into wine during aging in a barrel. One of the main oaklins, 11-guaiacylcatechinpyrylium, was also detected in a commercial table red wine aged in oak barrels. Reaction with furfural is also reported [97–99].

### 3.6. Cacao and Coffee

Cocoa and coffee beans contain proanthocyanidins and caffeoyl esters, respectively. Cocoa beans (the seeds of the *Theobroma cacao*) are processed for chocolate manufacturing *via* fermentation and the roasting process [100]. The cocoa beans contain catechins and dimeric to oligomeric procyanidins with 4–8 or 4–6 inter-flavan linkages. In addition, the presence of A-type procyanidins with 4–8 and 2-O-7 linkages and their glycosides were reported [101].

**Figure 14.**
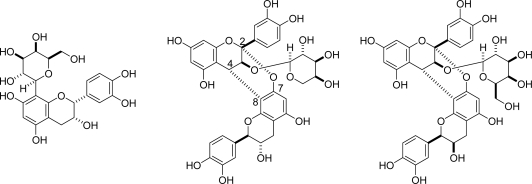
Structures of catechin and procyanidin glycosides isolated from cocoa liquor [102].

From the cocoa liquor produced from fermented and roasted cocoa beans, the catechin C-glycoside and A-type procyanidins glycosides were isolated [102] (Figure 14). During the processing, the proanthocyanidins decreased and were perhaps insolubilized, however, the chemical mechanism is not clear.

**Scheme 14.**
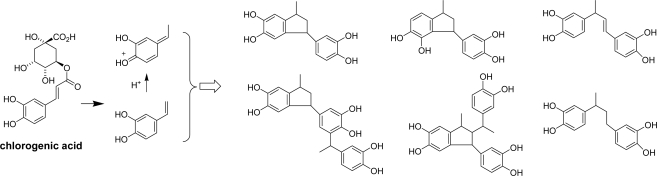
Decomposition of chlorogenic acid upon roasting of coffee beans [103].

Roasting of coffee beans is an interesting issue from the view point of food processing. The major phenolic constituent is chlorogenic acid and related hydroxycinnamoyl quinic acids. Frank *et al*. demonstrated that caffeoyl esters are decomposed by roasting to give 4-vinylcatechol units [103] (Scheme 14). Oligomerization of the C_6_–C_2_ units yielded the unique 1,3-bis(3′,4′-dihydroxyphenyl) butane, *trans*-1,3-bis(3′,4′-dihydroxyphenyl)-1-butene, and hydroxylated phenylindanes. These substances are all related to the bitter-taste of coffee.

## 4. Conclusions

The reason why plants accumulate polyphenols is thought to be related to the plant defense system [104–106], and the functions of the polyphenols depend on their chemical reactivity and physicochemical properties. The structural diversity of plant polyphenols in nature suggests that polyphenols have many different and wide-ranging functions. Some polyphenols, such as catechins and proanthocyanidins, are susceptible to enzymatic and non-enzymatic oxidation depending on the plant. Polyphenol oxidation in plant tissues, as observed in black tea production, proceeds along with a reduction in oxygen molecules or polyphenol quinones. Reactivity of these quinones with proteins and other coexisting compounds [61] also plays a significant role during the post harvesting period. The secondary polyphenols produced in plants after physical damage of the tissue is probably related to the plant defense system, though many of the products have not been characterized chemically. Artificial processing including drying, fermentation and roasting, are different from the normal reactions such as insolubilization and polymerization occurring in living plants and thus, produce different compounds. Recent scientific studies have confidently indicated that polyphenols in foods have various health benefits and thus, it continues to be important to identify mechanisms of their production and their chemical structures.
